# Supplementary material for: Cross-layer transmission realized by light-emitting memristor for constructing ultra-deep neural network with transfer learning ability
Source: Nat Commun. 2024 Mar 2;15:1930. doi: 10.1038/s41467-024-46246-3 (PMC10908859; doi:10.1038/s41467-024-46246-3)
Supplement: Supplementary file 1 — Supplementary Information [file 41467_2024_46246_MOESM1_ESM.pdf]

Supplementary Information

Cross-layer Transmission Realized by Light-emitting Memristor for Constructing Ultra-deep Neural Network with Transfer Learning Ability

Zhenjia Chen[1,2], Zhenyuan Lin[1,2], Ji Yang[3], Cong Chen[1,2], Di Liu[1,2], Liuting Shan[1,2], Yuanyuan Hu[4], Tailiang Guo[1,2], Huipeng Chen[*,1,2]

[1]Institute of Optoelectronic Display, National & Local United Engineering Lab of Flat Panel Display Technology, Fuzhou University, Fuzhou 350002, China

[2]Fujian Science & Technology Innovation Laboratory for Optoelectronic Information of China, Fuzhou 350100, China

[3]College of Computer and Data Science, Fuzhou University, Fuzhou, Fujian, China

[4]Changsha Semiconductor Technology and Application Innovation Research Institute, College of Semiconductors (College of Integrated Circuits), Hunan University, Changsha 410082, China.

*****Corresponding author. Email addresses: hpchen@fzu.edu.cn

Methods

Realization of hardware-software co-design neural network

The neural network model was built by hardware-software co-design, and the software part was deployed on FPGA (Xilinx AXU3EGB). First, after the input image was calculated by the input layer and the hidden layer 1, five outputs were generated, which were converted into corresponding voltages by DAC and input into the memristor array. Since the conductance of memristors cannot be negative, the conductance difference between two parallel memristors was used to represent the weight, and the two memristors were respectively connected in series with diodes in opposite directions to facilitate writing and reading conductance. The conductance of each device was adjusted to one of the selected 15 conductance states by using a continuous pulse of 1ms and 5 V. After that, the converted five voltage signals were input into the circuit, and the current of the device was read by controlling the switch. After ADC conversion, it was transmitted to the hidden layer 2 of FPGA for calculation, and finally the identification result was obtained. In the photodiode array circuit, photodiodes are used to receive equivalent signals, which were converted by ADC and then entered FPGA for identification.

Construction of UPENN

In the construction of UPENN, we first designed two kinds of cross-layer transmission bottleneck structures, namely BTNK 1 and BTNK 2, and set the first layer convolution result of each BTNK 1 as cross-layer information to simulate the optical and electrical output of the device. This information has three purposes, one

was for layer-by-layer transmission, the other was to accumulate the results after convolution processing and layer-by-layer transmission as the final output of the bottleneck structure and entered the next structure. Third, the information was directly transmitted to the last BTNK 2 of this Stage for accumulation operation. Finally, a neural network with 53 convolution layers and a fully connected layer was realized, and the network included the corresponding pooling layer, BN layer and 8 cross-regional transmission layers.

The structure of Contrast Net was like UPENN, but all the cross-layer transmission parts were removed, and only the network framework of layer-by-layer transmission was retained.

Construction of USRNN

In the construction of USRNN, we first designed ClBlock with BTNKs. Furthermore, the first-layer convolutional results within each ClBlock are designated to carry inter-layer information, simulating the light output and electrical output of the device. These information serve three purposes: firstly, for inter-layer transmission; secondly, to add the convolutional results to the output of the ClBlock as the final output of BTNK, which then enters the next ClBlock. Thirdly, to carry the convolutional results across 15 ClBlocks and all ClBlocks, and to accumulate them with the results of the 16th and the last ClBlock, thereby preventing gradient vanishing and exploding. Finally, the variation in the number of layers in the upsample module enables the realization of USRNN with 133 and 135 layers.

Pre-learning and Re-learning of Network

1. The models of two networks were built by Python, used ImageNet dataset in the pre-learning process, and obtained network weights. After that, we selected eight different data sets on Kaggle to verify the migration learning ability of the networks. Firstly, the test set of each dataset was used to test the two networks directly, and the corresponding accuracy was obtained. Then, the neural network was retrained with the trainset of 8 datasets, and the network was evaluated with the test set. Because UPENN has obtained good network parameters in the pre-training, in the re-learning, we frozen most of the network structures, and only allowed the last BTNK structure and the full connection layer to adjust. As for Contrast Net, we still allow it to train for the whole network to see if it can get better results.

2. The training of the model with a 2x resolution restoration structure in USRNN begins using the training set from the DIV2K dataset. Upon completion of this training phase, the weights of all ClBlocks are frozen, and the Upsample module is modified for 4x resolution restoration before proceeding with further training.

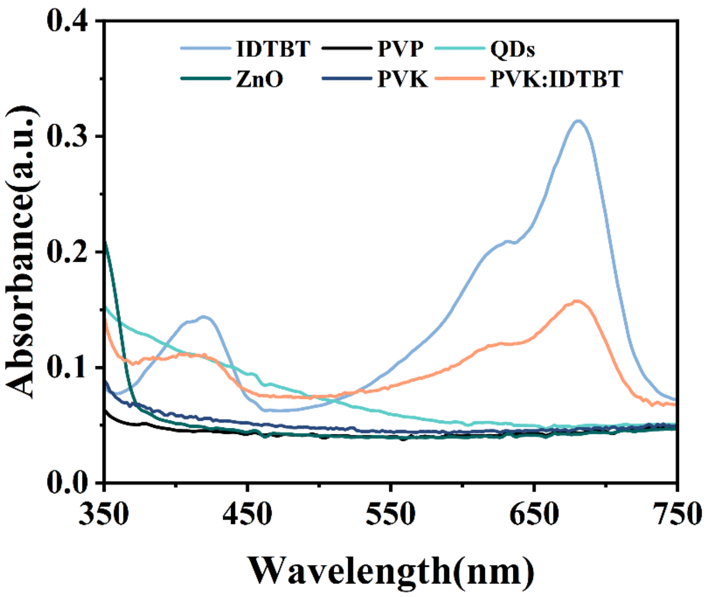

Figure. S1 Ultraviolet-visible absorption spectra of materials in devices.

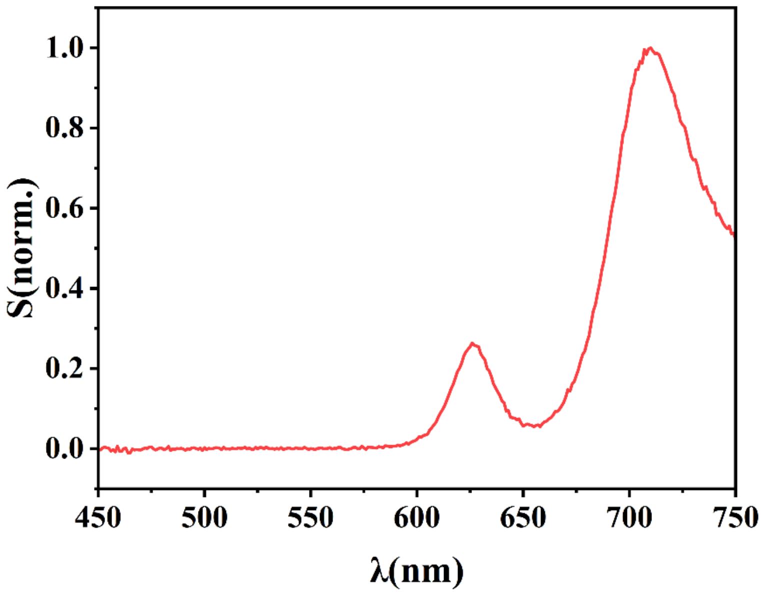

Figure. S2 The emission spectrum of light-emitting memristor.

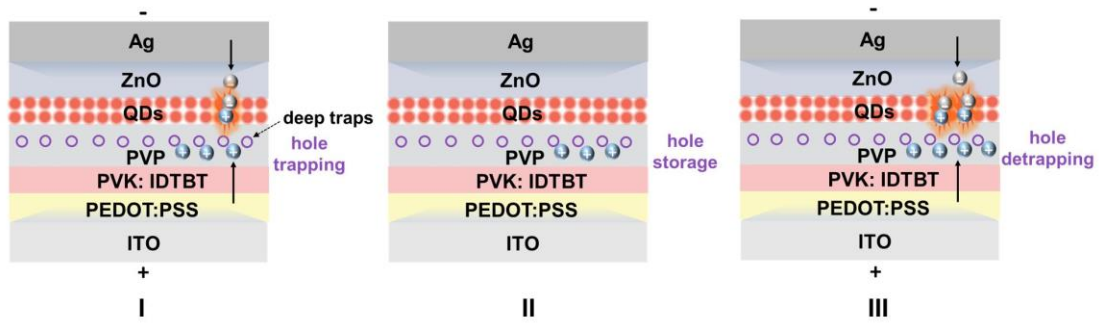

Figure. S3 The working mechanism of PVP charge trapping layer. Part of the holes can be captured by the PVP layer when bias is applied (I). Then, when the bias is removed, the holes are stored in the PVP layer (II). When bias is applied again, trapped holes are released under the action of an applied electric field (III), thus increasing the conductivity and brightness of the device and achieving a simulation of synaptic plasticity.

Polymer poly (4-vinyl phenol) (PVP) is a dielectric material, the polar groups contained in the PVP side group contain enormous amount of deep traps that allow charging and decharging carriers upon applied voltage. Therefore, in the previous report, this is the main reason for the hysteresis of OFETs with PVP as a dielectric layer [*Appl. Phys. Lett. 89, 262120 (2006)*; *Appl. Phys. Lett. 93, 143302 (2008)*; *Appl. Phys. Lett. 108, 173301 (2016)*]. Here, we exploit this feature to achieve the characteristics of artificial synapses by embedding capture layer PVP in QLED. Part of the holes can be captured by the PVP layer when bias is applied (I). Then, when the bias is removed, the holes are stored in the PVP layer (II). When bias is applied again, trapped holes are released under the action of an applied electric field (III), thus increasing the conductivity and brightness of the device and achieving a simulation of synaptic plasticity.

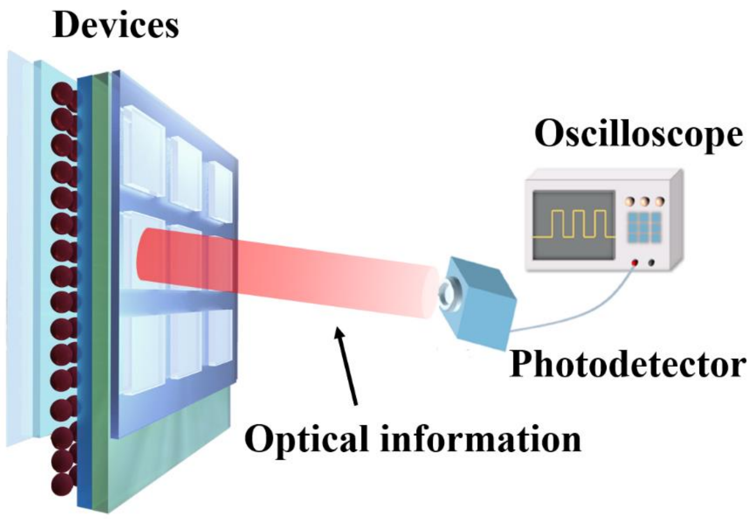

Figure. S4 Schematic diagram of recording EPSB signal with oscilloscope and photodetector.

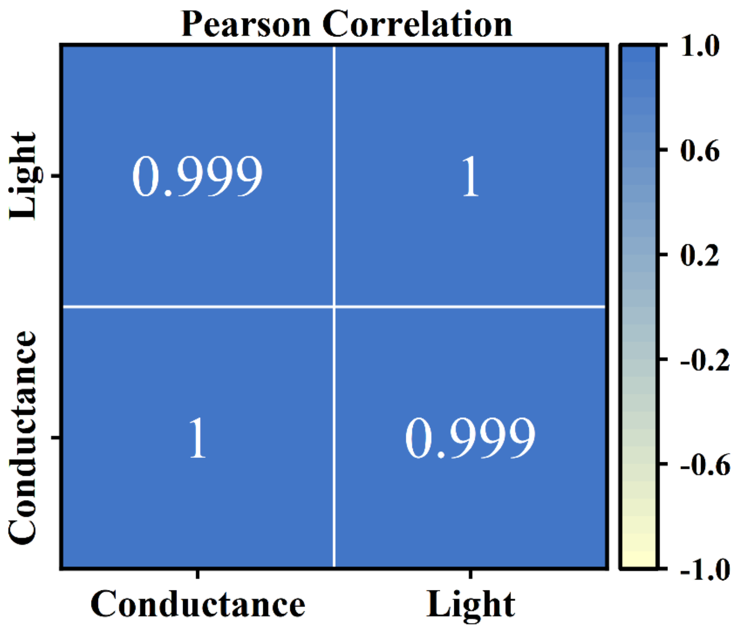

Figure. S5 Pearson correlation analysis of postsynaptic current and postsynaptic brightness.

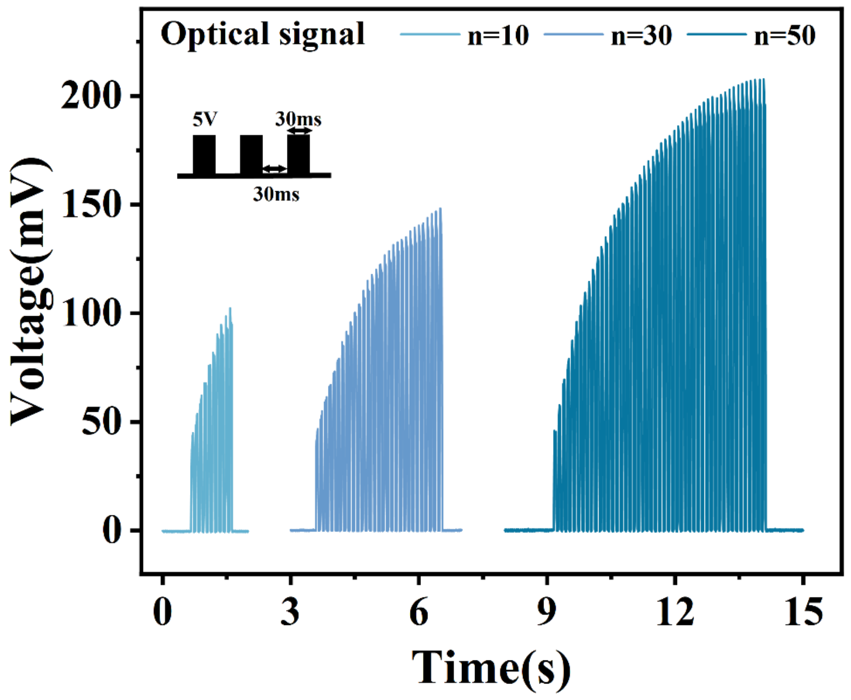

Figure. S6 The EPSBs caused by n = 10, n = 30 and n = 50 pulses.

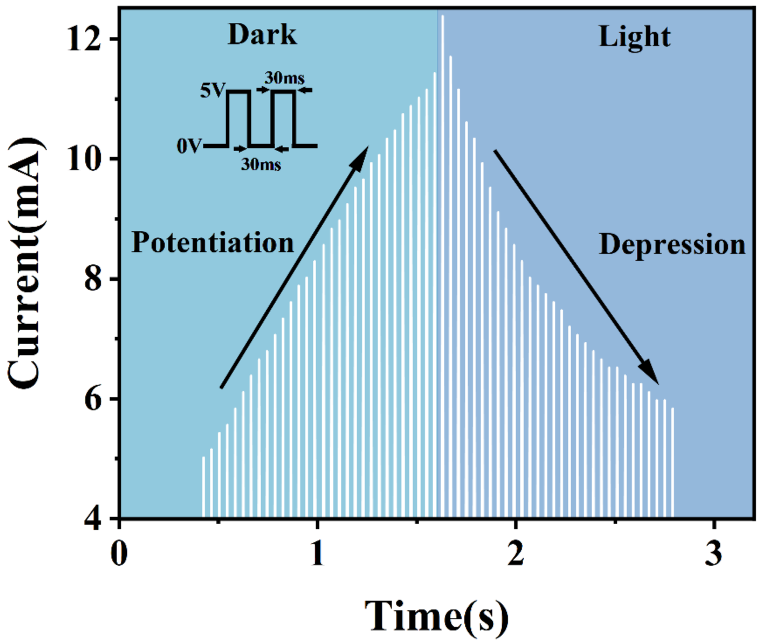

Figure. S7 The conductance enhancement and suppression curves obtained by applying continuous electrical pulse stimulation and UV stimulation.

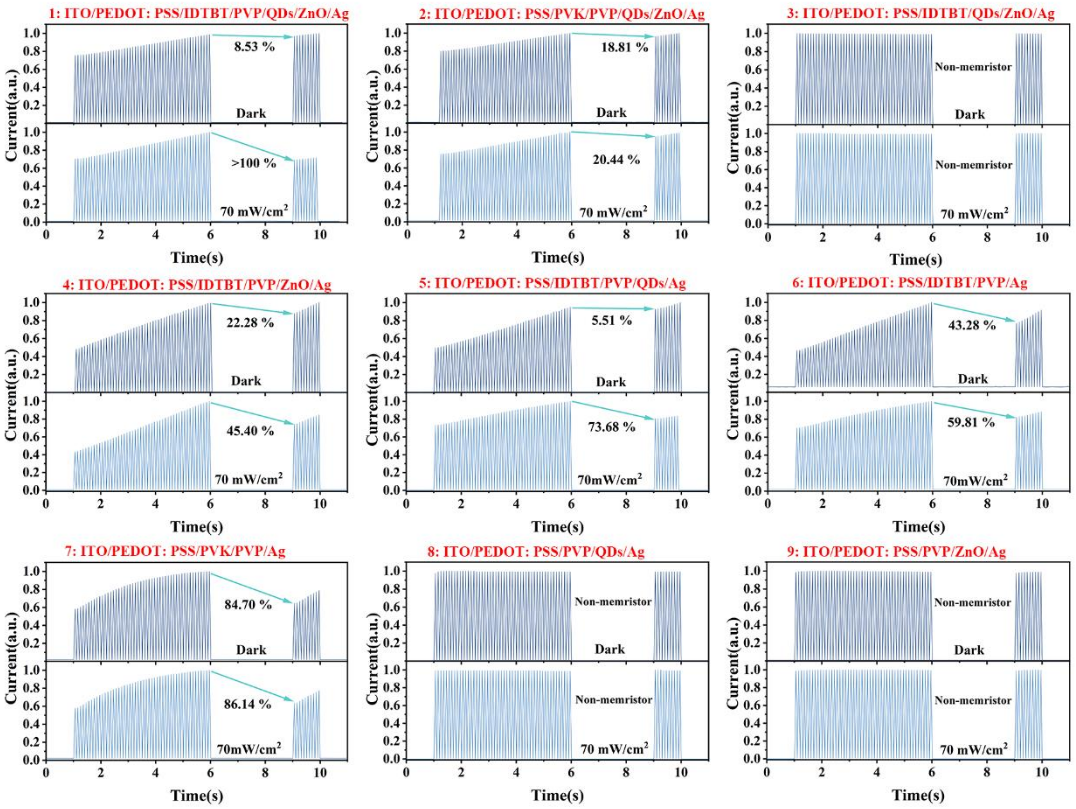

Figure. S8 The current variation graphs of the 9 devices. By applying 50 continuous 5 V electrical pulses with an interval of 50 ms, followed by a 3 s exposure to both dark and ultraviolet light conditions, then the electrical pulse stimulation was administered again.

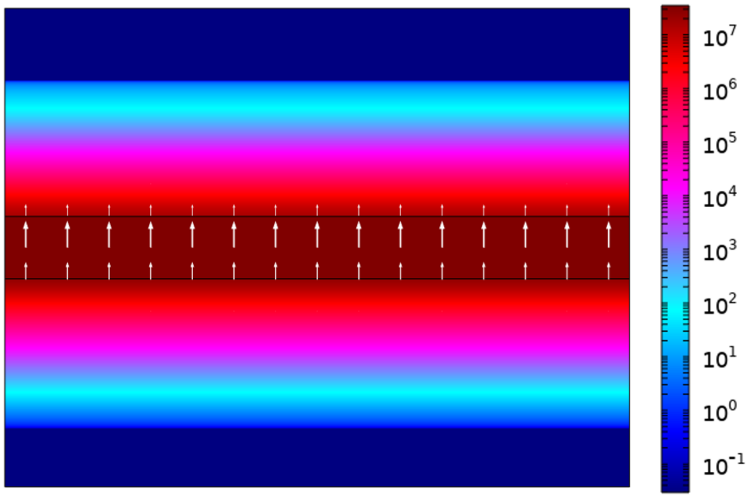

Figure. S9 Schematic diagram of built-in electric field distribution generated by device under UV simulated by software, with arrow as electric field direction and scale unit as V/m.

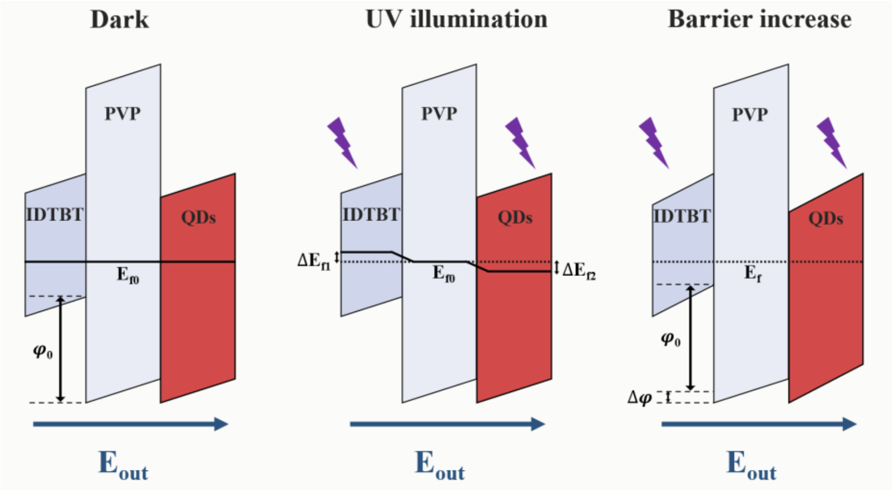

Figure. S10 Schematic diagram of barrier change caused by Fermi energy level change.

In the dark state, the Fermi level of the device is in equilibrium. Due to UV stimulation, a large number of electron-hole pairs are generated in IDTBT, but the hole concentration hardly changes, and the electron concentration changes greatly. According to Fermi Dirac distribution function, the increase of electron concentration leads to the increase of the Fermi level of materials(ΔE_{f1}), and the Fermi level between materials is rebalanced, which leads to the increase of the potential barrier ($\Delta\varphi$) and further hinders the hole transmission.

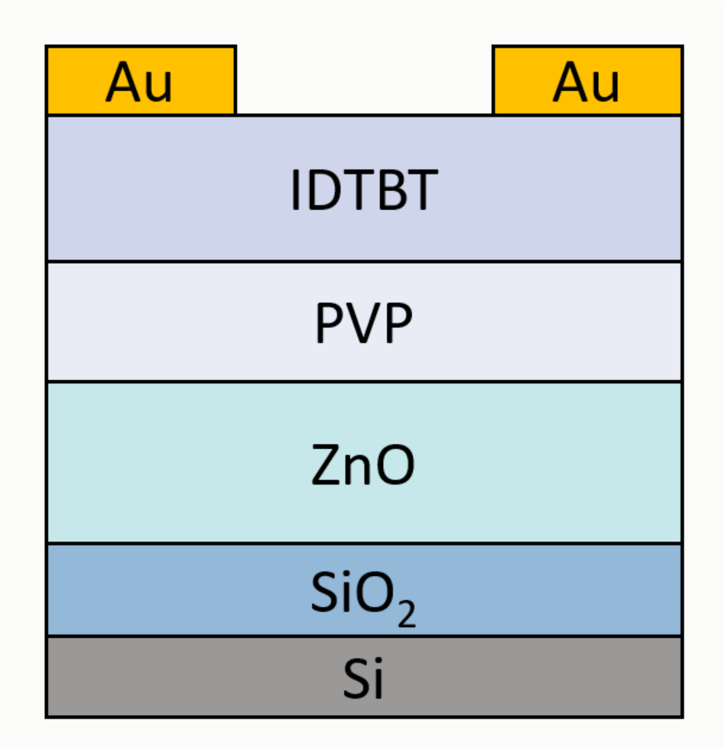

Figure. S11 Schematic diagram of organic field effect transistor with PIN structure.

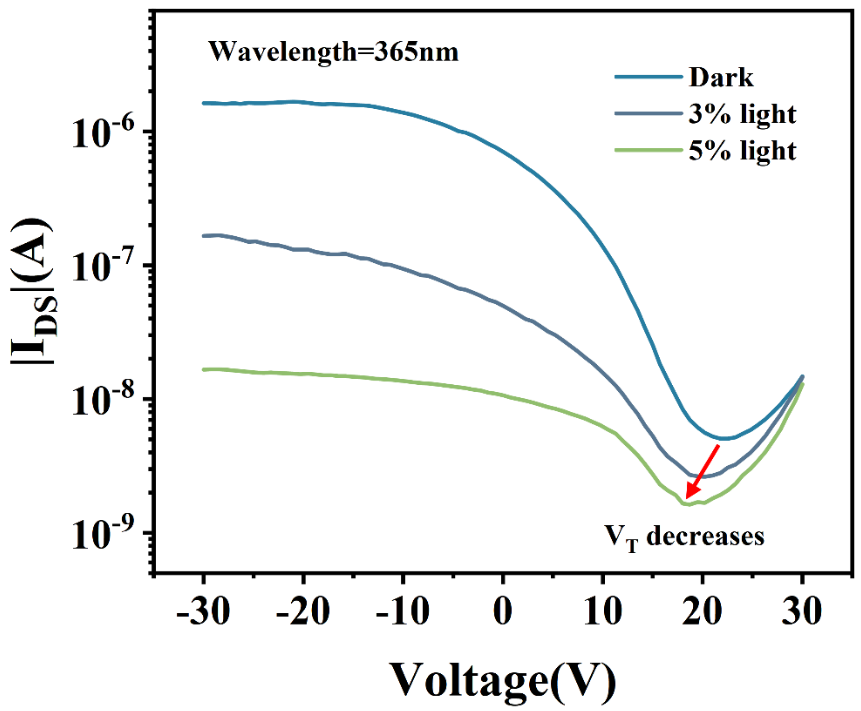

Figure.12 The I-V curves of organic field effect transistor with and without UV light. The optical power of 3% and 5% is 1 mW/cm^2 and 3 mW/cm^2 respectively.

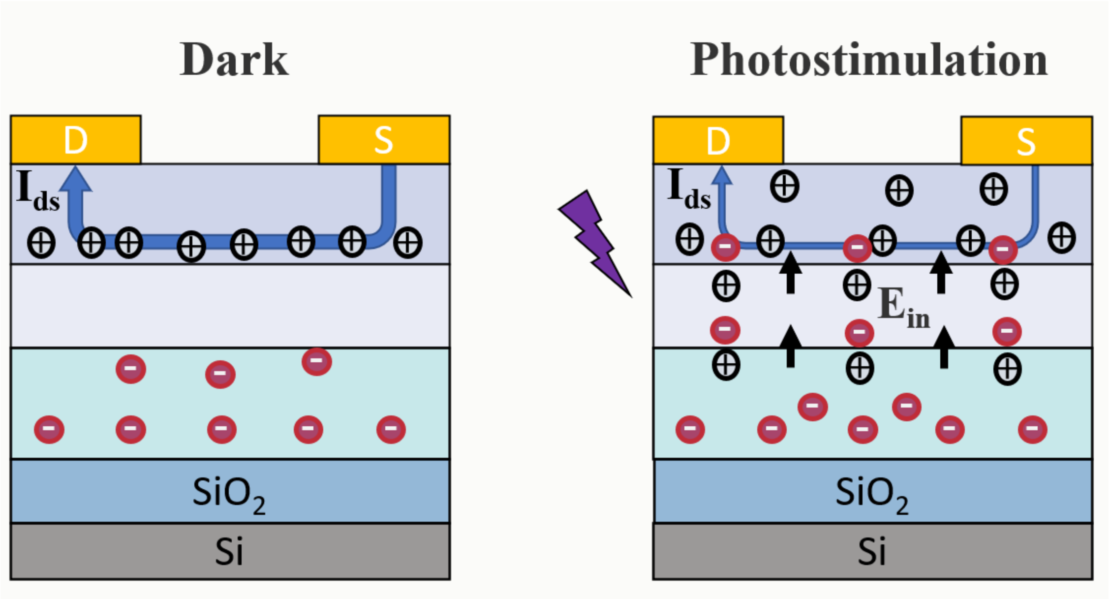

Figure. S13 Schematic diagram of photo-induced electric field and conductance change of OTFT.

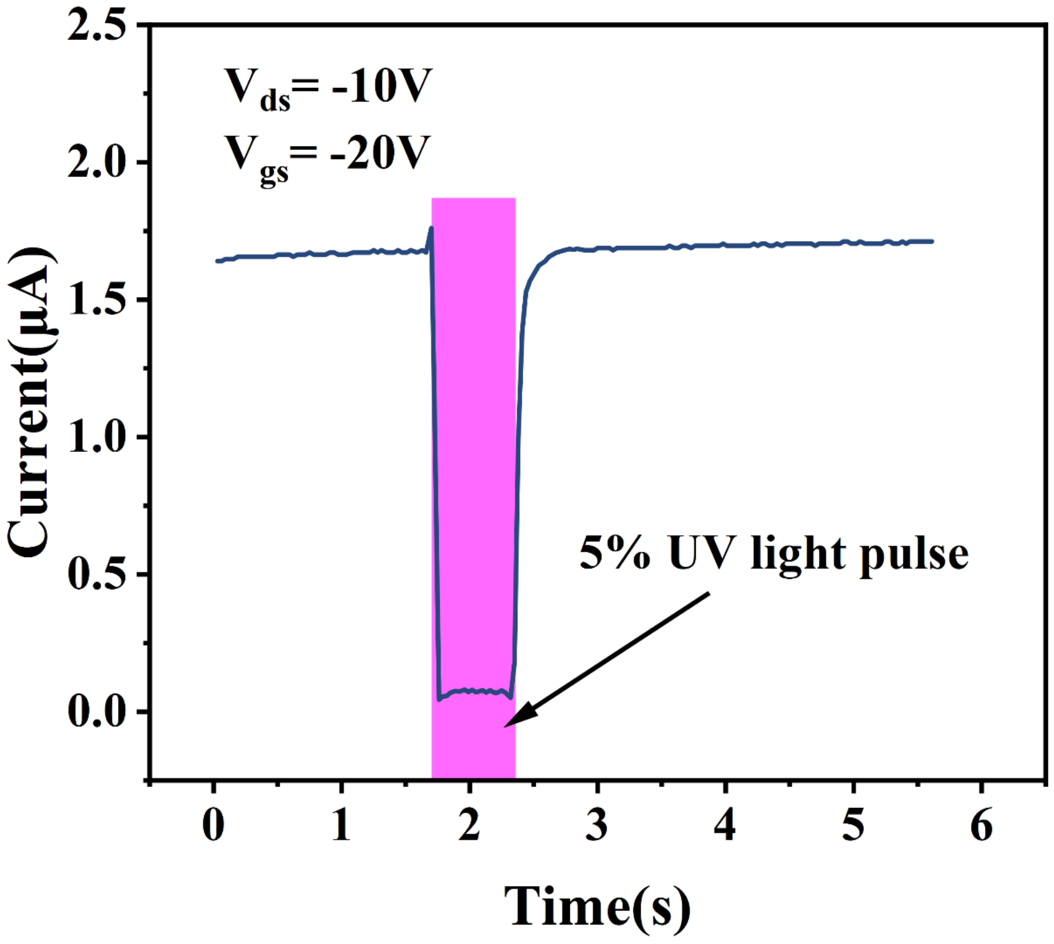

Figure. S14 The current reduction of transistor under UV irradiation.

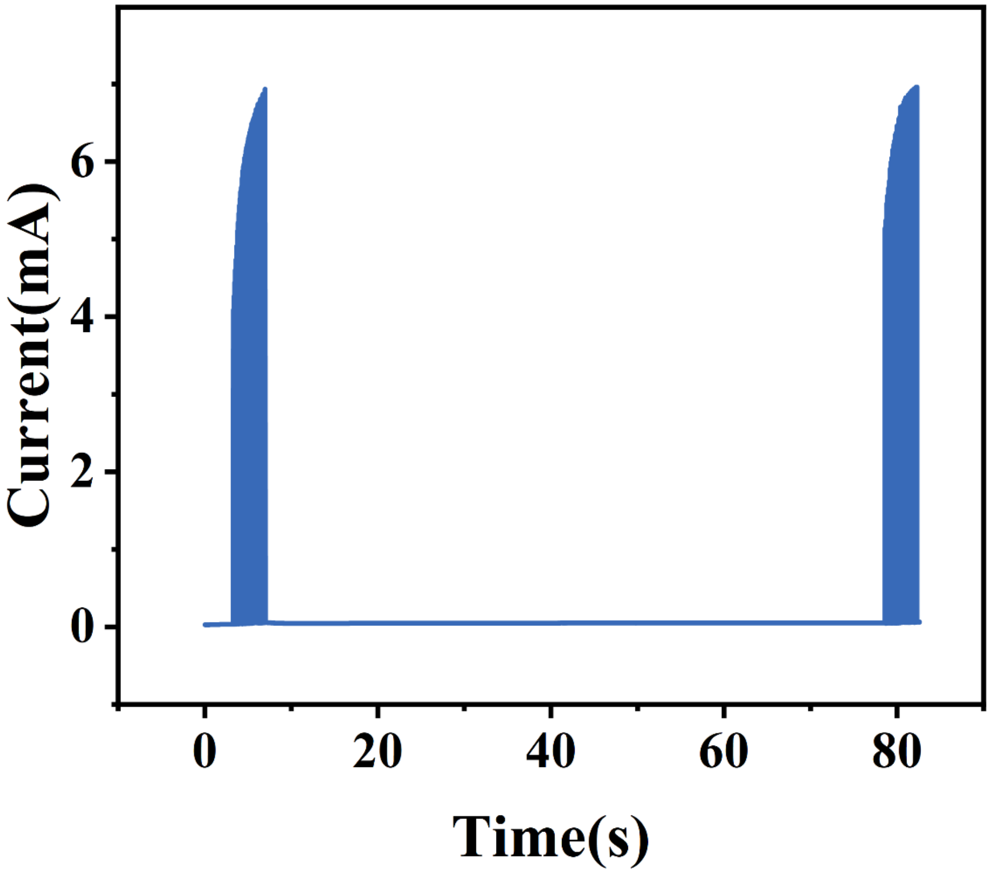

Figure. S15 The decay time required by light-emitting memristor after 50 impulses stimulation.

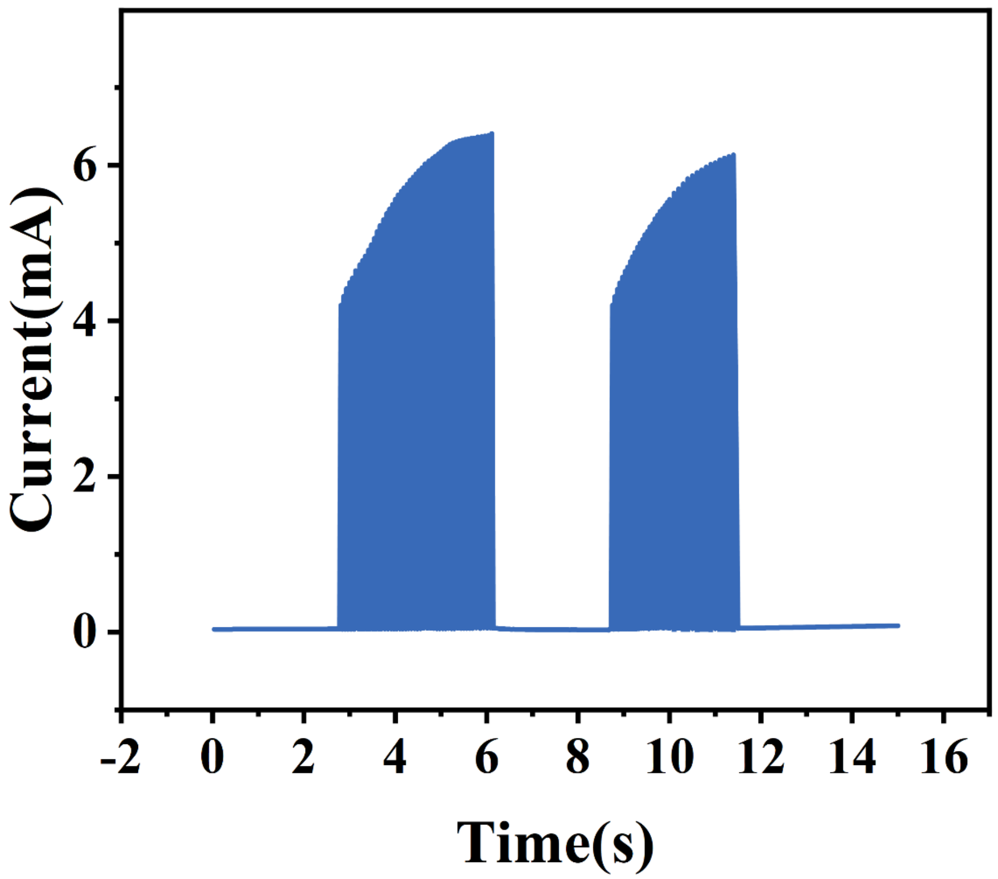

Figure. S16 Schematic diagram of resetting weight of light-emitting memristor after 50 pulse stimulation by UV irradiation.

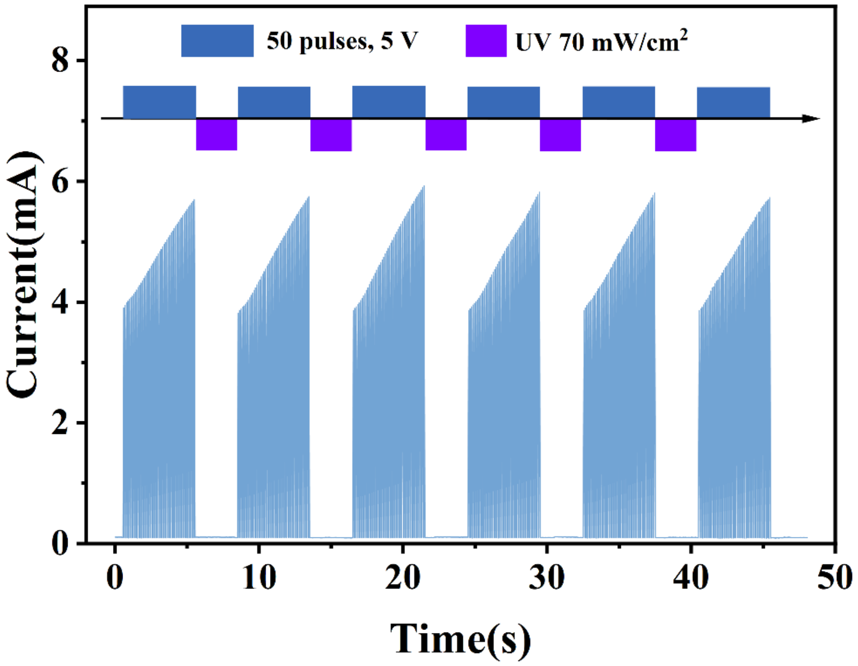

Figure. S17 Multi-cycle repetition of device reset operation after 50 pulse electric pulses.

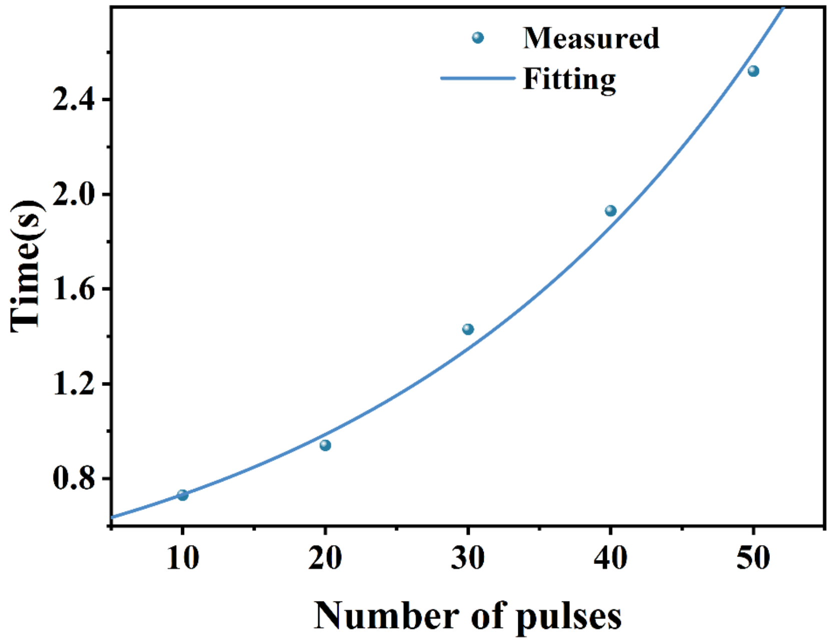

Figure. S18 Lighting time required for resetting with different pulse numbers (at 70 mW/cm^2).

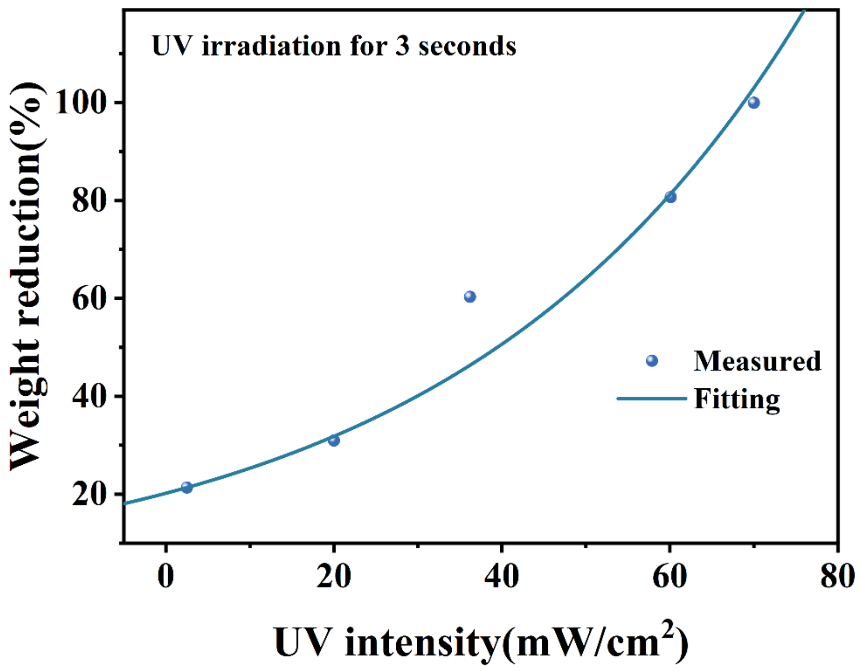

Figure. S19 Weight reduction data under different optical powers after 50 electric pulses.

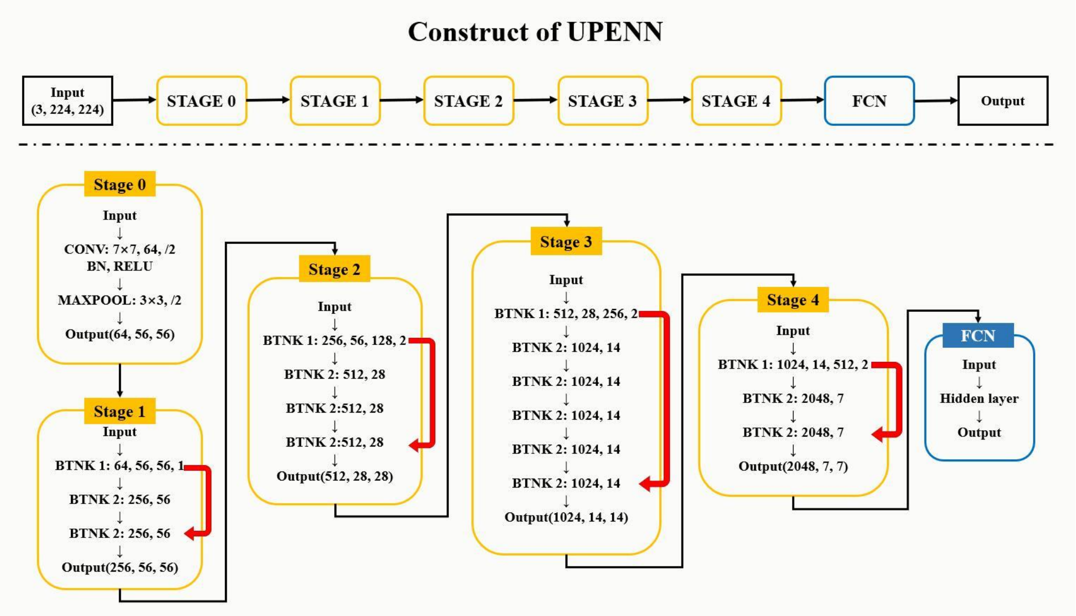

Figure. S20 Detailed structural diagram of UPENN. Stage 1 to Stage 4 are different structures of ClBlock. ClBlock consists of BTNK 1 and BTNK 2, where BTNK 1 is the transmitting end of the optical interlayer signal, and BTNK 2 is the receiving end of the optical interlayer signal. BTNK 2, which is not indicated by the red arrow, does not receive optical signals crossing over the stage.

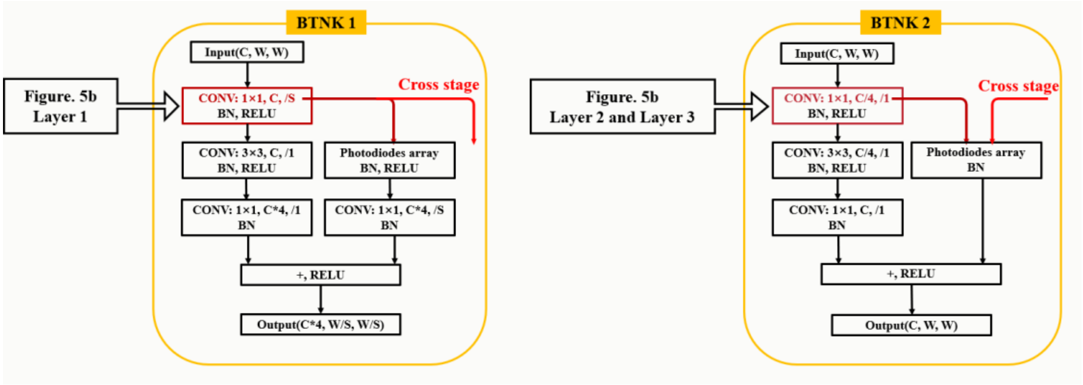

Figure. S21 The structural schematic diagram of BTNK used in UPENN and the position of gradient distribution obtained by calculation in Figure 5b.

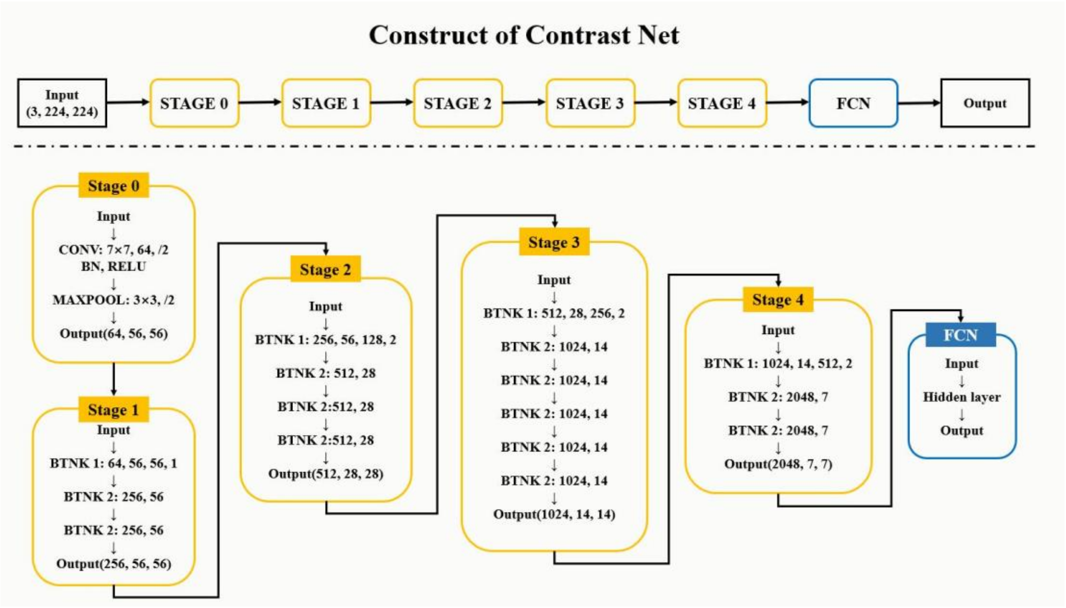

Figure. S22 Detailed structural diagram of Contrast Net.

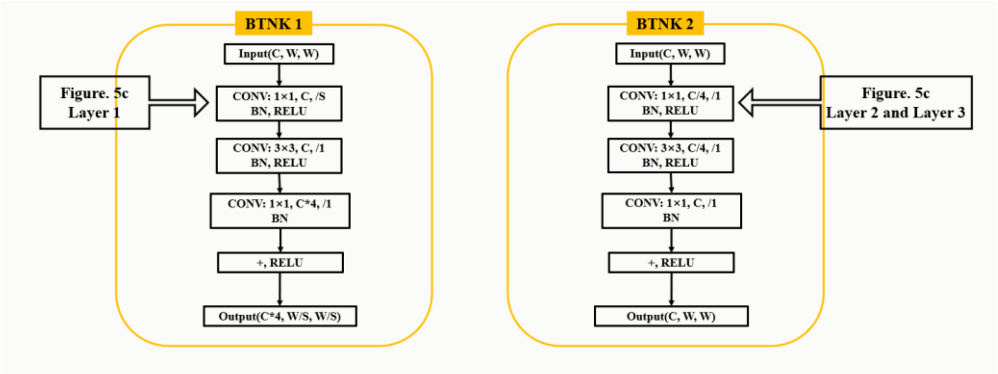

Figure. S23 The structural schematic diagram of BTNK used in Contrast Net and the position of gradient distribution obtained by calculation in Figure 5c.

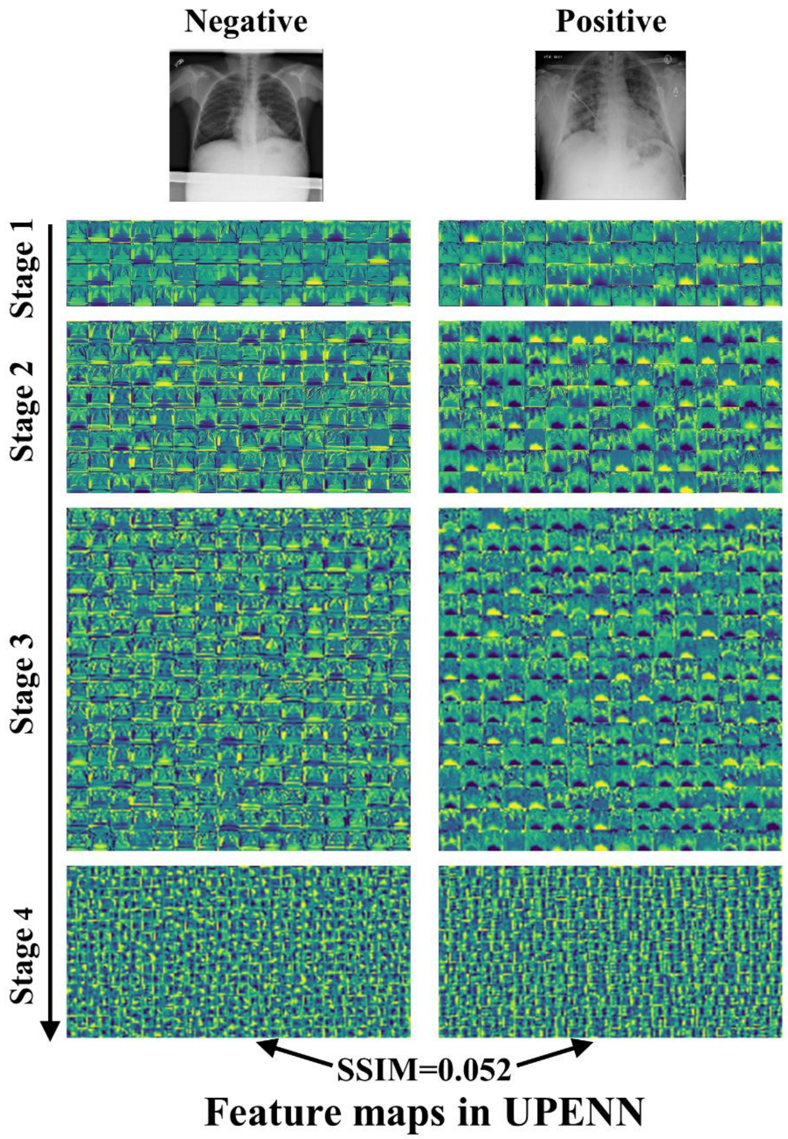

Figure. S24 The feature maps of two X-ray images in UPENN and the SSIM of two feature maps in stage 4. (The feature maps are obtained after the first convolution in each stage of the network.)

Figure. S19 shows the feature maps obtained from stage 1 to stage 4 in UPENN for the X-ray images labeled as negative and positive in a binary classification task. In an ideal scenario, the neural network should extract completely different features for negative and positive cases to achieve close to 100% accuracy in distinguishing all images into two categories. It is evident from the figure that the two images acquire different features under the influence of the same convolutional kernels (the convolutional kernels are consistent at each position of the feature map), particularly in stage 3 and stage 4. This indicates that UPENN can extract features by successfully avoiding gradient vanishing through cross-layer transmission and obtaining effective convolutional kernels through pre-training. By using SSIM to analyze the similarity between the two feature maps in stage 4, the SSIM coefficient is only 0.052, indicating that the two maps are extremely dissimilar. Therefore, UPENN is capable of effectively distinguishing between negative and positive cases.

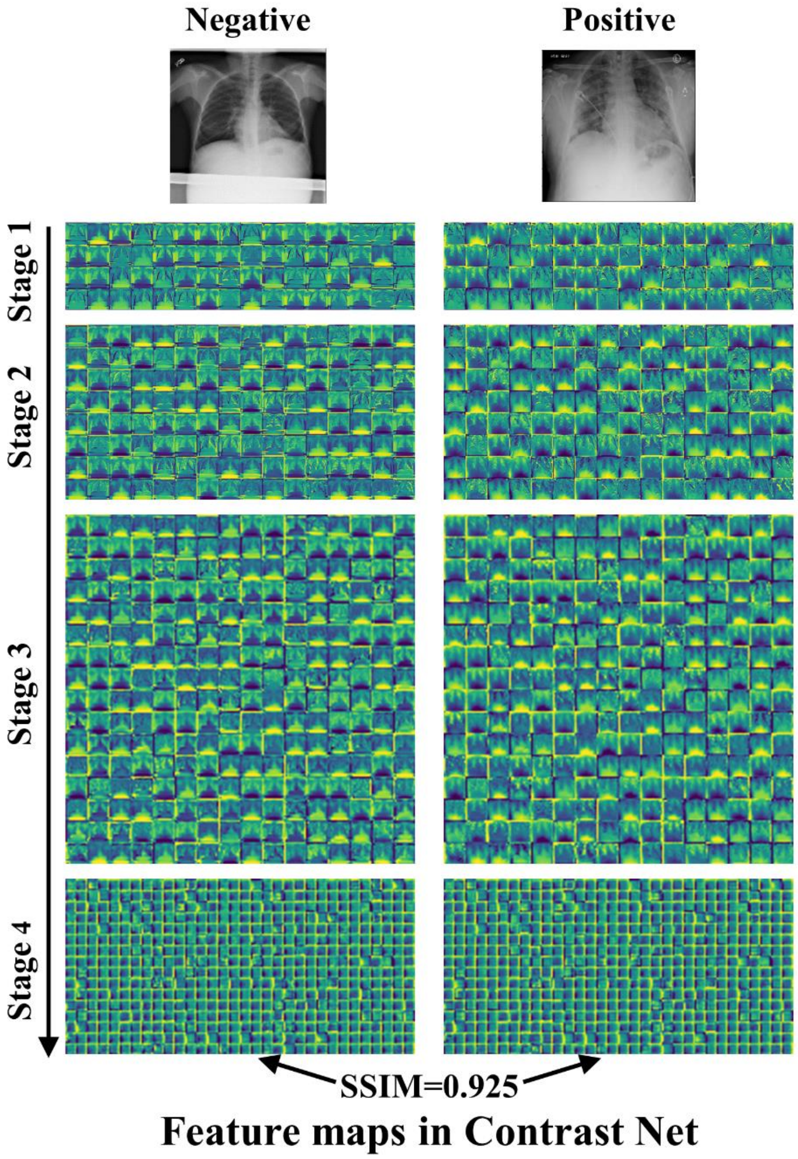

Figure. S25 The feature maps of two X-ray images in Contrast Net and the SSIM of two feature maps in stage 4. (The feature maps are obtained after the first convolution in each stage of the network.)

Figure. S20 shows the feature maps obtained from stage 1 to stage 4 in Contrast Net for the X-ray images labeled as negative and positive in a binary classification task. In an ideal scenario, the neural network should extract completely different features for negative and positive cases to achieve close to 100% accuracy in distinguishing all images into two categories. It is obvious from the figure that the two images have obtained very similar features under the influence of the same convolution kernel. This indicates that the convolution kernel of Contrast Net cannot effectively distinguish the image features of the two cases, which is essentially due to the invalid convolution kernel obtained in the pre-learning process caused by the gradient vanishing. By using SSIM to analyze the similarity between the two characteristic graphs in Stage 4, the SSIM coefficient is 0.925, which indicates that the two graphs are very similar. Therefore, Contrast Net cannot effectively distinguish negative and positive cases.

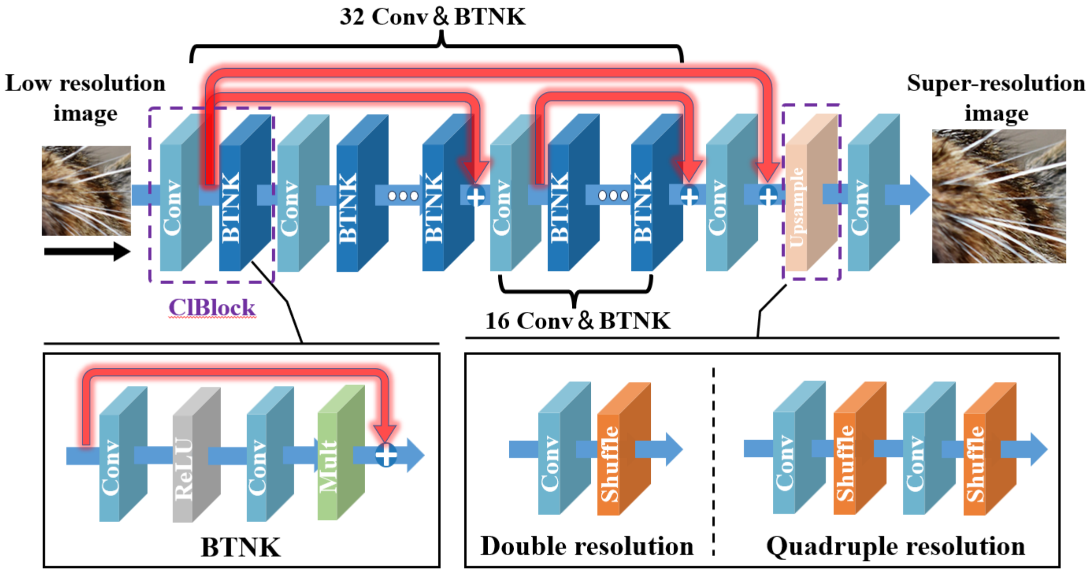

Figure. S26 The ClBlock for constructing USRNN and structure of USRNN.

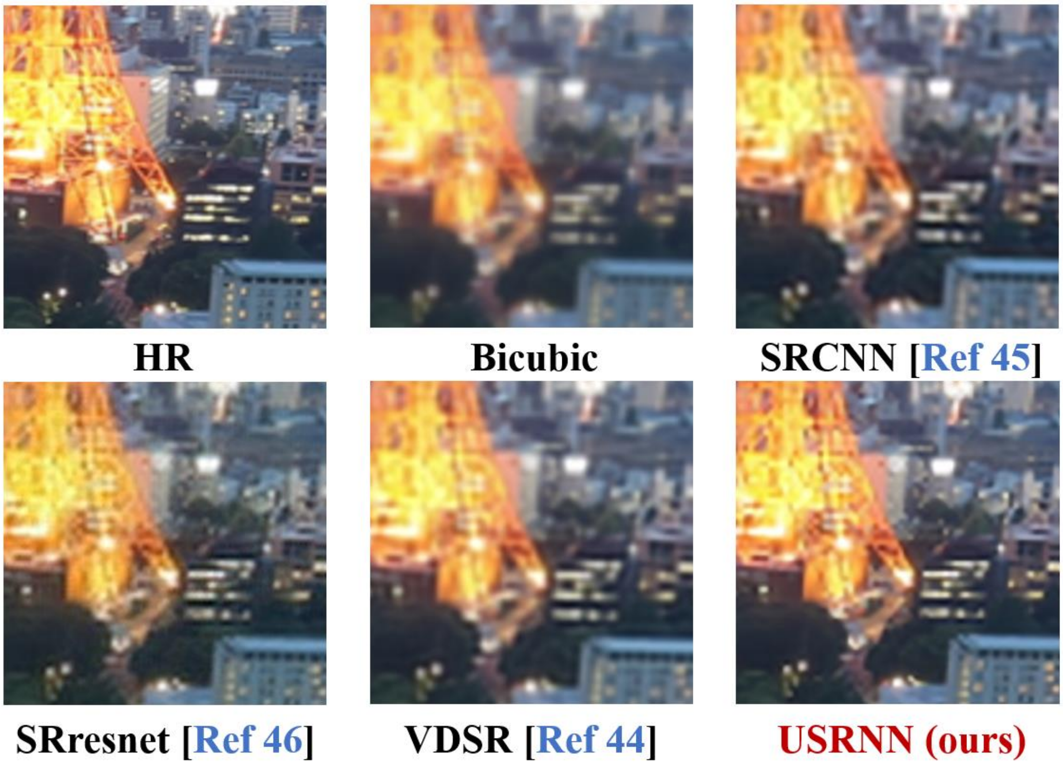

Figure. S27 Partial repair details of Figure. 6b.

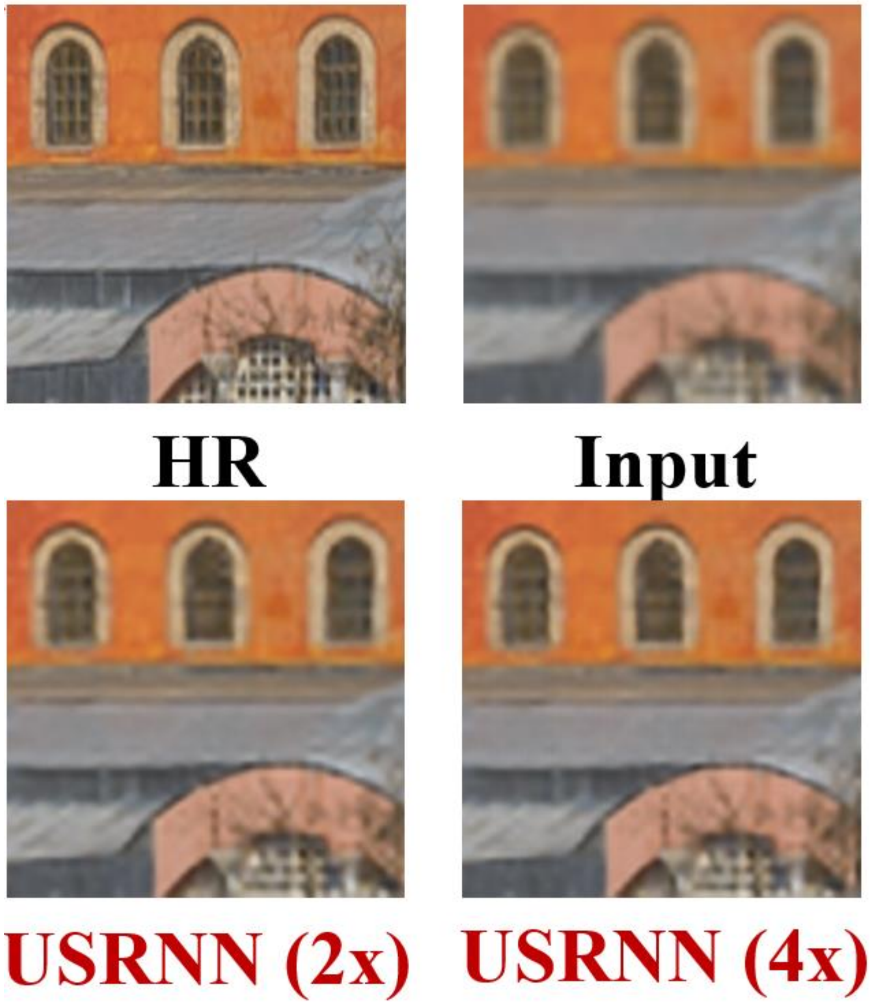

Figure. S28 Partial repair details of Figure. 6c.

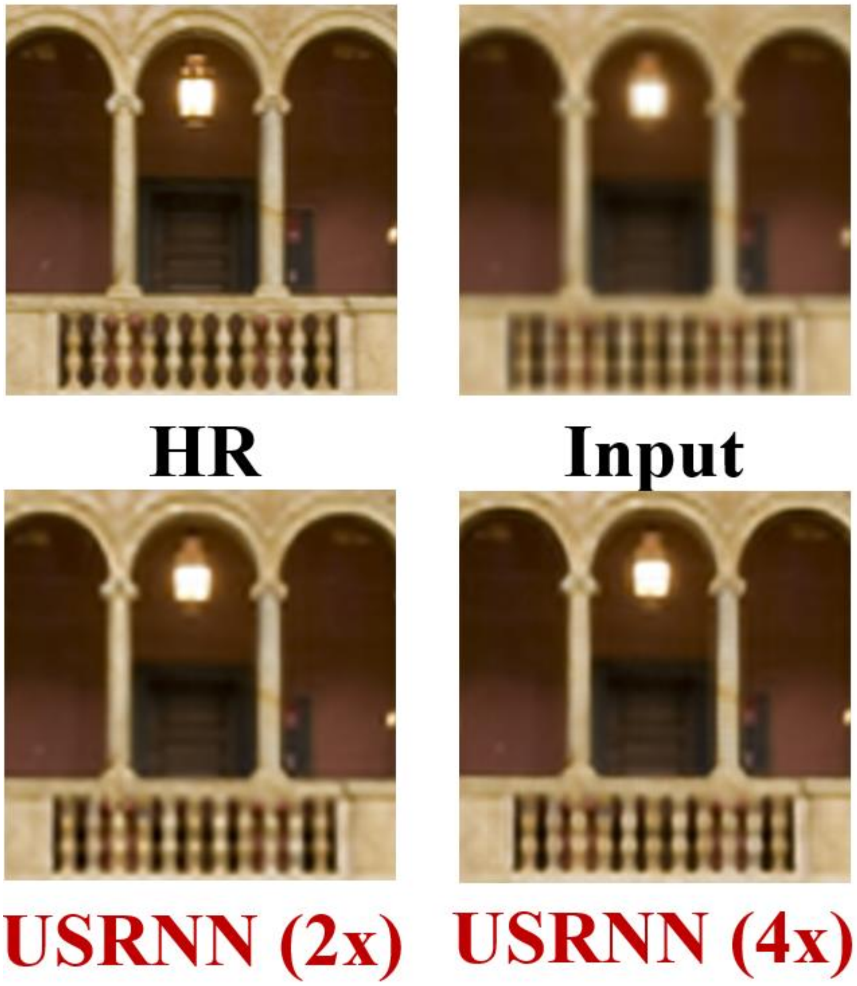

Figure. S29 Partial repair details of Figure. 6d.

Supplementary Table 1

Table of UV negative photoconductive characteristics of various structures.

**Number**	**Structure**	**UV negative photoconductivity (Yes/No)**
**0**	ITO/PEDOT/PVK:IDTBT/PVP/QDs/ZnO/Ag	yes
**1**	ITO/PEDOT/IDTBT/PVP/QDs/ZnO/Ag	yes
**2**	ITO/PEDOT/PVK/PVP/QDs/ZnO/Ag	no
**3**	ITO/PEDOT/IDTBT/QDs/ZnO/Ag	no
**4**	ITO/PEDOT/IDTBT/PVP/ZnO/Ag	yes
**5**	ITO/PEDOT/IDTBT/PVP/QDs/Ag	yes
**6**	ITO/PEDOT/IDTBT/PVP/Ag	no
**7**	ITO/PEDOT/PVK/PVP/Ag	no
**8**	ITO/PEDOT/PVP/QDs/Ag	no
**9**	ITO/PEDOT/PVP/ZnO/Ag	no

Supplementary Table 2

Table of accuracies obtained by UPENN and Contrast Net from 8 datasets after pre-learning and re-learning.

Dataset	Number of species	Accuracy (%)			
		Contrast Net		UPENN	
		Pre-learning	Relearning	Pre-learning	Relearning
**Covid-19 chest X-Ray images**	2	50.3	50.4	94.3	98.2
**Brain Tumor MRI Dataset**	4	27.0	27.1	97.0	98.0
**Dogs & Cats Images**	2	50.1	50.2	94.5	97.2
**Butterfly & Moths Images**	100	1.51	1.50	92.6	95.8
**Intel Image Scene**	6	17.8	17.5	91.1	93.2
**Sports balls**	15	9.0	8.60	79.9	85.1
**Cards Image**	53	2.1	2.20	77.5	81.1
**Tree Nuts**	10	13.1	13.0	66.8	77.5
Average accuracy		21.36	21.31	86.71	90.76

Supplementary Note 1

OTFT with negative photoconductive effect

The current-voltage (I-V) transfer curves of the device are measured under dark conditions and UV irradiation (**Figure. S12**). The V_{ds} applied to the device is -20V, and the V_{gs} is scanned from 30V to -30V. The device presents a typical P-type transfer curve. Under irradiation, the turn-on voltage VT of the device shifted to the left due to the electric field generated by electrons and holes at the interface. This implies that the positive V_{gs} required to turn off the device decreased, indicating the presence of an electric field Ep at the interface between the gate and the channel (**Figure. S13**). Since the channel of the transistor is formed at the interface (**Figure. S13**), the conductance of the channel formed by hole decreases under the influence of the Ep, as observed in the I-V curve where the saturation current of the device decreases (**Figure. S12**). Additionally, when negative V_{gs} and UV stimulation are applied, it can be observed that the current of the device briefly increases and then rapidly drops (**Figure. S14**). This is because the concentration of channel carriers increases momentarily upon illumination, but then a photovoltaic electric field E_p opposite to E_{gs} is generated at the IDTBT/PVP interface, causing the channel carriers of the device to be repelled.

Supplementary Note 2

Equation describing the gradient change of DNN with or without cross-layer transmission structure

The disappearance (exploding) of gradient is one of the fundamental reasons for the low accuracy of DNN. Assuming that $X_{i,}$ X_{i+1} are the input and output of a shallow layer in Contrast Net (X_{i+1} is the input of the next layer), X_l is the output of the deep layer, F() is the intra-layer mapping, and the activation function is r(), then:

$$X_{i+1} = \mathrm{r}\big(\mathrm{F}(X_i, W_i)\big) \qquad (1)$$

The derivative of X_{i+1} to X_i is:

$$\frac{\partial X_{i+1}}{\partial X_i} = \mathrm{r}'_{X_i}(\mathrm{F}(X_i, W_i)) \cdot \mathrm{F}'_{X_i}(X_i, W_i) \qquad (2)$$

Write it down as:

$$\frac{\partial X_{i+1}}{\partial X_i} = \mathrm{r}'_i \cdot \mathrm{F}'_{X_i}(X_i, W_i) \qquad (3)$$

Similarly:

$$\frac{\partial X_I}{\partial X_{I-1}} = \mathrm{r}'_{I-1} \cdot \mathrm{F}'_{X_{I-1}}(X_{I-1}, W_{I-1}) \qquad (4)$$

When updating the gradient:

$$\frac{\partial Loss}{\partial X_i} = \frac{\partial Loss}{\partial X_I} \cdot \frac{\partial X_I}{\partial X_i} = \frac{\partial Loss}{\partial X_I} \cdot \frac{\partial X_I}{\partial X_{I-1}} \cdot \frac{\partial X_{I-1}}{\partial X_{I-2}} \cdots\cdot \frac{\partial X_{i+1}}{\partial X_i}$$

$$= \frac{\partial Loss}{\partial X_I} \cdot \frac{\partial X_I}{\partial X_i} \cdot \mathrm{r}'_{I-1} \cdot \mathrm{F}'_{X_{I-1}}(X_{I-1}, W_{I-1}) \cdot \mathrm{r}'_{I-2} \cdot \mathrm{F}'_{X_{I-2}}(X_{I-2}, W_{I-2}) \cdots\cdot \mathrm{r}'_i \cdot \mathrm{F}'_{X_i}(X_i, W_i)$$

$$= \frac{\partial Loss}{\partial X_I} \cdot \prod_{n=i}^{I-1} \mathrm{r}'_n \cdot \mathrm{F}'_{X_n}(X_n, W_n) \qquad (5)$$

From equation (5), it becomes evident that when the value of $\mathrm{r}'_n \cdot \mathrm{F}'_{X_n}(X_n, W_n)$ is lower than 1, the gradient of X_I tends to approach 0 during propagation through the layers of the network. Despite the ReLu activation function being chosen in the network to maintain r'_n constant equal to 1, the existence of $\mathrm{F}'_{X_n}(X_n, W_n)$ within this structure still causes the Contrast Net to experience the issue of gradient vanishing (or exploding).

In UPENN, optical signals are designed for the cross-layer transmission of identity. When using the ReLu activation function, the input X_i of the i layer and the output F() of the cross-layer transmission block are accumulated as the final output of the output block, that is:

$$X_{i+1} = \mathrm{r}\big(X_i + \mathrm{F}(X_i, W_i)\big) = X_i + \mathrm{F}(X_i, W_i) \qquad (6)$$

$$X_{i+2} = \mathrm{r}\big(X_{i+1} + \mathrm{F}(X_{i+1}, W_{i+1})\big) = X_{i+1} + \mathrm{F}(X_{i+1}, W_{i+1})$$

$$= X_i + \mathrm{F}(X_i, W_i) + \mathrm{F}(X_{i+1}, W_{i+1}) \qquad (7)$$

Similarly:

$$X_I = X_i + \sum_{n=i}^{I-1} \mathrm{F}(X_n, W_n) \qquad (8)$$

When updating the gradient:

$$\frac{\partial Loss}{\partial X_i} = \frac{\partial Loss}{\partial X_I} \cdot \frac{\partial X_I}{\partial X_i} = \frac{\partial Loss}{\partial X_I} \cdot \frac{\partial (X_i + \sum_{n=i}^{I-1} \mathrm{F}(X_n, W_n))}{\partial X_i}$$

$$= \frac{\partial Loss}{\partial X_I} \cdot (1 + \frac{\partial \sum_{n=i}^{I-1} \mathrm{F}(X_n, W_n)}{\partial X_i}) \qquad (9)$$

Based on equation (9), the presence of 1 allows the gradient of X_I to propagate across multiple layers to X_i, effectively mitigating the issue of gradient vanishing(exploding).
